# A Descriptive Study on the Association Between the Sensory Profile and the Autistic Quotient in Italian 3–12-Year-Old Preschoolers and Schoolers with Autism

**DOI:** 10.3390/bs16010139

**Published:** 2026-01-19

**Authors:** Annalisa Levante, Rosa Angela Fabio, Chiara Martis, Rossella Suriano, Valentina Romeo, Flavia Lecciso

**Affiliations:** 1 Department of Human and Social Sciences, University of Salento, 73100 Lecce, Italy; chiara.martis@unisalento.it (C.M.); flavia.lecciso@unisalento.it (F.L.); 2 Lab of Applied Psychology, Department of Human and Social Sciences, University of Salento, 73100 Lecce, Italy; 3 Department of Biomedical and Dental Sciences and Morpho-Functional Imaging, University of Messina, 98100 Messina, Italy; rosaangela.fabio@unime.it (R.A.F.); suriano.rossellaz@gmail.com (R.S.); valentina-romeo@hotmail.it (V.R.)

**Keywords:** autism, autistic-like trait severity, sensory reactivity, sensory profile, preschoolers, schoolers

## Abstract

Sensory reactivity has recently been introduced as a diagnostic criterion for autism, and growing attention is being paid to considering children’s behavioural responses to sensory stimuli. This study explored sensory reactivity in a sample of preschool- and school-aged autistic children. Parents of 68 participants [21 preschoolers (3–5 years) and 47 school-aged children (6–11 years)] completed an e-survey (Ethical Committee: 2024-412). Two research questions were addressed to explore: a. whether sensory reactivity dimensions differ according to autistic-like trait severity (medium vs. high) and b. whether sensory reactivity differs between preschool- and school-aged children. Controlling for age and sex, the results showed that children with higher autistic-like trait severity exhibited greater sensory reactivity across all dimensions. The interaction also supported higher sensory reactivity in each dimension for children with higher severity levels. In addition, no significant difference and interaction emerged between age group and sensory reactivity, supporting the potential stability of these features over time. However, group comparisons indicated that school-aged children showed higher parental-reported movement sensitivity, particularly during rough play or balance-related activities. These findings highlight the importance of considering sensory reactivity in autism diagnosis and in designing supportive and tailored intervention environments.

## 1. Introduction

Autism Spectrum Disorder (ASD) is characterized by difficulties in social communication and interaction, together with restricted interests and repetitive behaviours (APA, 2013, 2022/2023). In the previous revision of the *Diagnostic and Statistical Manual of Mental Disorders* (DSM-IV-TR; APA, 1994), difficulties in sensory processing were not explicitly included in the diagnostic criteria, although they were frequently reported (approximately 90%; e.g., Baranek et al., 2006) as individual characteristics (Kern et al., 2007; Rogers et al., 2003; Tavassoli et al., 2014; Tomchek & Dunn, 2007). For example, the comparative study by Tomchek and Dunn (2007) on preschoolers with and without autism found that most children with autism showed heightened sensory reactivity in the domains of Under-Responsive/Seeks Sensation, Auditory Filtering, and Tactile Sensitivity, compared with typically developing peers.

Based on accumulating evidence, the fifth edition of the *Diagnostic and Statistical Manual of Mental Disorders* (DSM-5; APA, 2013) and its subsequent text revision (APA, 2022/2023) included sensory reactivity as part of Criterion B (i.e., restricted, repetitive patterns of behaviour, interests, or activities). Specifically, sensory reactivity is described in terms of hyper- or hypo-reactivity to environmental sensory stimuli, such as apparent indifference to pain or temperature, adverse responses to specific sounds or textures, excessive smelling or touching of objects, and visual fascination with lights or movement (APA, 2013, p. 50). Hyper-reactivity refers to an excessive or aversive response to sensory input, often experienced as overwhelming, whereas hypo-reactivity refers to reduced responsiveness or apparent indifference to sensory stimuli.

As highlighted by a meta-analysis (Ben-Sasson et al., 2009) and several empirical studies (Lane et al., 2010, 2011, 2014; Mayer, 2017), the severity of autistic-like traits appears to be associated with variability in behavioural responses to sensory stimuli. In other words, different levels of autistic-like traits may relate to sensory reactivity in distinct ways. For instance, Mayer (2017), examining autistic adults, assessed autistic-like traits using the Autism Quotient in relation to sensory profiles and found that the domains of attention to detail and imagination were not significantly associated with total sensory profile scores, whereas social skills, attention switching, and communication showed significant associations. In contrast, studies by Lane and colleagues (Lane et al., 2010, 2011, 2014) on autistic children indicated that sensory subtypes are better characterized by modality and responsiveness rather than overall severity. Thus, the relationship between autistic-like traits and sensory profiles does not appear to increase linearly with severity but rather shows substantial heterogeneity across individuals.

Atypical sensory reactivity, including both hyper- and hypo-reactivity, may lead autistic individuals to experience difficulties in regulating and organizing the type and intensity of their behavioural responses to environmental sensory stimuli (Ben-Sasson et al., 2009; Bogdashina, 2016). Consequently, behavioural responses following sensory exposure may be dysfunctional, distressing, or inefficient (Tomchek & Dunn, 2007), thereby limiting environmental participation and engagement (Baranek et al., 2006; Leekam et al., 2007) and potentially increasing social isolation (Cosbey et al., 2010; Parham et al., 2007). Importantly, investigating sensory reactivity in early childhood may also play a crucial role in preventing comorbid conditions (Fabbri-Destro et al., 2022). For example, internalizing problems may emerge when atypical sensory responses lead to persistent distress. In this regard, Horder et al. (2014) found that greater sensory reactivity was associated with higher levels of anxiety.

The far-reaching impact of sensory reactivity on daily participation and everyday activities highlights the need for further investigation of this domain. Supporting this perspective, a recent opinion paper by Narzisi et al. (2025) emphasized the importance of addressing sensory reactivity within autism research and clinical practice. Despite its relevance, sensory reactivity remains relatively underexplored. While longitudinal studies are essential for evaluating the effectiveness of targeted clinical interventions—aimed at reducing both autistic-like traits and maladaptive sensory-related behavioural responses—descriptive studies are also necessary to inform research and clinical practice about which sensory dimensions warrant greater attention. Accordingly, the present study aimed to contribute to the existing literature by examining sensory reactivity in autism, offering insights into the sensory experiences of individuals with varying levels of autistic-like traits. These findings may also have practical implications, as they can support professionals in designing training and recreational activities that take individual sensory sensitivities into account.

## 2. Materials and Methods

### 2.1. Study’s Purposes

Due to the explorative nature of the current study, two research questions (RQs) are formulated:

**RQ1:**  *What dimensions of sensory reactivity are significantly different in autistic preschoolers and schoolers, according to the two degrees of autistic-like traits’ severity (medium versus high)? What is the interacting effect of the severity degree of the autistic-like traits (medium versus high) on the sensory reactivity dimensions after controlling for the child’s age and sex?*

**RQ2:**  *What dimensions of sensory reactivity are significantly different in the preschoolers and school-age autistic children? What is the interacting effect of the group (preschoolers versus schoolers) on sensory reactivity after controlling for the child’s sex?*

### 2.2. Study Design and Procedure

The cross-sectional and descriptive study was conducted in Italy between September 2024 and June 2025, using an e-survey imported into Google Forms and disseminated via major social media platforms (e.g., WhatsApp and Facebook). All procedures complied with the principles of the Declaration of Helsinki, and the study was approved by the University of Messina’s Ethical Committee before data collection (protocol code, 2024-412; 16 May 2024).

The inclusion criteria for sampling are as follows: (1) being over 18 years old; (2) having an autistic child aged 3–11 years; and (3) being fluent in the Italian language. Before completing the e-survey, participants read the spreadsheet regarding their privacy and rights and signed an e-consent. In addition, participants were informed that they could withdraw from the e-survey at any moment without explanation. No compensation, voucher, or payment was provided to participate in the study.

### 2.3. Study Sample

Sixty-eight parents of 3–5-year-old preschoolers (n = 21; 15 males and 6 females) and 6–11-year-old schoolers (n = 47; 37 males and 10 females) completed the e-survey. The parents’ mean age is 41.1 (SD = 6.43) years. Mothers completed the e-survey in the majority of cases (n = 60).

### 2.4. Study’s Measures

#### 2.4.1. Autistic-like Traits Assessment

The Italian version (Ruta et al., 2012) of the Autism Spectrum Quotient—Children’s Version (AQ-Child; Auyeung et al., 2008) was administered. It is a parent-report questionnaire assessing the degree of the severity of the autistic-like traits in preschoolers and schoolers (3–11 years). Importantly, it is not a diagnostic tool, but it provides an estimate of how closely a child’s behaviour aligns with autistic-like characteristics.

The questionnaire consists of 50 items rated on a 4-point Likert scale ranging from “*Definitely Agree*” to “*Definitely Disagree*.” In accordance with the scoring system by Baron-Cohen et al. (2001), items are scored as 1 for a response in the autistic-like direction and 0 for a non-autistic-like response.

A total score is calculated as the sum of all items, and it theoretically ranges from 0 to 50. In addition, five domains related to autism and the broader phenotype are calculated as a sum: social skills (items 1, 11, 13, 15, 22, 36, 44, 45, 47, and 48; item example: “*S/he prefers to do things with others rather than on her/his own*”; theoretical range = 0–20), attention switching (items 2, 4, 10, 16, 25, 32, 34, 37, 43, and 46; item example: “*S/he prefers to do things the same way over and over again*”; theoretical range = 0–20), attention to detail (items 5, 6, 9, 12, 19, 23, 28, 29, 30, and 49; item example: “*S/he often notices small sounds when others do not*”; theoretical range = 0–20), communication (items 7, 17, 18, 26, 27, 31, 33, 35, 38, and 39; item example: “*S/he has difficulty understanding rules for polite behaviour*”; theoretical range = 0–20), and imagination (items 3, 8, 14, 20, 21, 24, 40, 41, 42, and 50; item example: “*If s/he tries to imagine something, s/he finds it very easy to create a picture in her/his mind*”; theoretical range = 0–20).

Both for the total score and the 5 domains, the higher the scores, the more autistic-like the behaviour. For the current study, the total score was used.

The Italian version of the questionnaire shows reliable cross-cultural (Ruta et al., 2012) and discriminative validity (Aiello et al., 2021) in distinguishing different phenotypes.

#### 2.4.2. Sensory Reactivity Assessment

The Italian version (Nale et al., 2017) of the Short Sensory Profile (Dunn, 1994) was administered. It is a parent-report questionnaire designed to assess children’s responses to everyday sensory experiences and stimuli.

The SSP consists of 38 items rated on a 5-point Likert scale ranging from “Never” (1) to “Always” (5). A higher score indicates a high individual reactivity to sensory stimuli.

A total score is calculated as the sum of all items, and it ranges from 38 to 190. In addition, seven dimensions of sensory reactivity are calculated as a sum: Tactile Sensitivity (TAS; items 1–7; item example: “*Expresses distress during grooming*”; theoretical range: 0–35); Taste/Smell Sensitivity (TSS; item 8–11; item example: “*Avoids certain tastes or food smells that are typically part of children’s diets*”; theoretical range: 0–20); Movement Sensitivity (MOV; items 12–14; item example: “*Becomes anxious or distressed when feet leave the ground*”; theoretical range: 0–15); Underresponsive/Seeks Sensation (USS; items 15–21; item example: “*Enjoy strange noises/seeks to make noise for noise’s sake*”; theoretical range: 0–30); Auditory Filtering (AF; item 22–27; item example: “*Has trouble completing tasks when the radio is on*”; theoretical range: 0–30); Low Energy/Weak LEW; (items 28–32; item example: “*Seems to have weak muscles*”; theoretical range: 0–25); and Visual/Auditory Sensitivity (VAS; item 33–38; item example: “*Holds hands over ears to protect ears from sound*”; theoretical range: 0–30).

Both for the total score and the 7 dimensions, the higher the scores, the more sensory reactivities. For the current study, the total score was used in the ANCOVA, and the score of each dimension was used in the group comparison analyses.

The Italian version of the questionnaire shows reliable cross-cultural validity (Nale et al., 2017; Valagussa et al., 2022).

### 2.5. Statistical Plan

The normality and homogeneity of the data were calculated. No group comparisons according to the children’s sex were computed due to the sex discrepancy. The associations between the degree of the severity of the autistic-like traits, sensory reactivity dimensions, and the children’s ages were analyzed using Spearman correlation analysis. Due to the descriptive nature of the current study, the two research questions have been tested using the ANCOVA to compare the sensory reactivity total score between the degree of severity of the autistic-like traits classification (medium versus high), firstly, and between age groups (preschoolers versus schoolers) in the second stage. In both models, the sensory reactivity total score was considered as the dependent variable, while the degree of the severity of the autistic-like traits classification (medium versus high) and the age groups (preschoolers versus schoolers) were considered as the categorical independent variables in the first and second models, respectively. ANCOVA provides the interacting effects after controlling for the possible effects of the children’s age and sex variables.

The significance threshold was set at 5%. Statistical analysis was performed using the IBM Statistical Package for Social Sciences [IBM SPSS, version 25].

## 3. Results

### 3.1. Preliminary Results

Shapiro–Wilk W is significant for both the autistic-like traits total score (W = 0.97; *p* = 0.006) and sensory reactivity total score (W = 0.93; *p* < 0.001), indicating that the data are not distributed normally. The homogeneity of variance reported no significant difference for the autistic-like traits total score [F(1,138) = 1.85; *p* = 0.176] and a significant one for the sensory reactivity total score [F(1,138) = 32.17; *p* < 0.001]. Although normality and homogeneity assumptions were not fully met, ANCOVA is considered robust to moderate violations of these assumptions in small-to-medium samples. Therefore, ANCOVA was retained as the primary analytic approach (Blanca Mena et al., 2017; Keselman et al., 2008). Nonparametric Mann–Whitney tests were additionally conducted to confirm the robustness of the results.

Regarding data collected in the present paper, the Cronbach alphas for the Autism Spectrum Quotient—Children’s Version are the following: Total score: α = 0.80; social skills: α = 0.70; attention switching: α = 0.70; attention to detail: α = 0.71; communication: α = 0.70; and imagination: α = 0.71.

On the Short Sensory Profile, the Cronbach alphas of the collected data are as follows: total score: α = 0.95; Tactile Sensitivity: α = 0.83; Taste/Smell Sensitivity: α = 0.93; Movement Sensitivity: α = 0.84; Underresponsive/Seeks Sensation: α = 0.81; Auditory Filtering: α = 0.87; Low Energy/Weak LEW: α = 0.93; and Visual/Auditory Sensitivity: α = 0.85.

### 3.2. Severity of Autistic-like Traits and Sensory Reactivity

The autistic-like trait severity has been calculated according to the autistic quotient total score, with a score of 32 or higher indicative of a clinically significant degree of autistic-like traits (Baron-Cohen et al., 2001). Similarly to other studies (A. E. Robertson & Simmons, 2013; C. E. Robertson & Baron-Cohen, 2017), three degrees of autistic-like trait severity were calculated: a low degree, ranging from 0 to 18; a medium degree, ranging from 19 to 31; and lastly, the high degree, ranging from 32 to 50.

In the current study, two children (all males) reported a low degree of autistic-like traits; 38 children (n = 30 males; n = 8 females) presented a medium degree of autistic-like traits; while 30 children (n = 22 males; n = 8 females) have been classified as reporting a high degree of autistic-like traits. Note that, due to the small number of children who were classified with a low degree of autistic-like traits (n = 2), they have been removed from the study’s analyses.

The severity of the sensory reactivity has been calculated according to the sensory profile total score, with a score of 141 or higher indicative of a clinically significant degree of sensory reactivity (McIntosh et al., 1999). Similarly to other studies (O’Donnell et al., 2012), for the current study, three degrees of severity of the sensory reactivity were calculated: a low degree, ranging from 38 to 129; a medium degree, varying from 130 to 140; and lastly, a high degree, ranging from 141 to 190.

In the current study, 49 children (n = 38 males; n = 11 females) reported a low degree of sensory reactivity; 9 children (n = 6 males; n = 2 females) presented a medium degree of sensory reactivity; while the 12 children (n = 10 males; n = 2 females) were classified as reporting a high degree of sensory reactivity.

Table 1 reports the frequencies of autistic children in the two degrees of severity of autistic-like traits and of sensory reactivity. Profiling children according to the degree of severity is not adequate due to the heterogeneity among the groups [χ^2^ = 16.785; *p* > 0.001].

### 3.3. Correlations

Except for the associations between Taste/Smell Sensitivity (TSS), Underresponsiveness/Seek Sensation (USS), and Low Energy/Weak (LEW), and the association between Movement Sensitivity (MOV) and Low Energy/Weak (LEW), Spearman correlations revealed significant and positive associations between the severity of the autistic-like traits and each dimension of sensory reactivity (see Table 2).

This means that the higher the autistic-like traits, the higher the sensory reactivity reported by parents. No associations between the children’s ages and the study’s variables were found.

### 3.4. Research Questions

**RQ1.**  *What dimensions of sensory reactivity are significantly different in autistic preschoolers and schoolers, according to the two degrees of autistic-like traits’ severity (medium versus high)? What is the interacting effect of the severity degree of the autistic-like traits (medium versus high) on the sensory reactivity dimensions after controlling for the child’s age and sex?*

**Figure 1:**
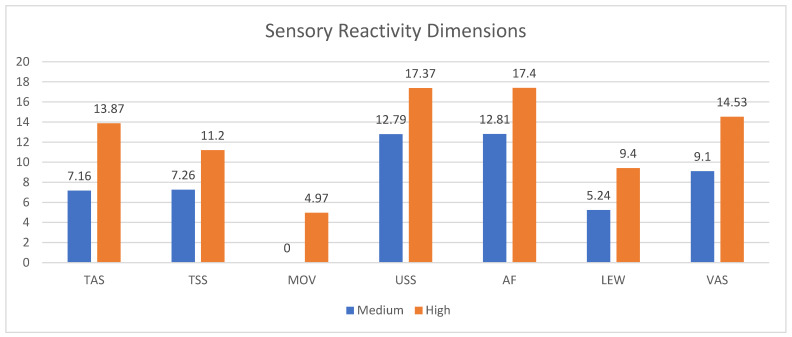
Sensory reactivity dimensions for autistic children with medium (blue) and high (orange) levels of autistic-like traits. Note: TAS: Tactile Sensitivity; TSS: Taste/Smell Sensitivity; MOV: Movement; USS: Underresponsive/Seeks Sensation; AF: Auditory Filtering; LEW: Low Energy/Weak; VAS: Visual/Auditory Sensitivity.

The results of group comparisons showed that all the dimensions of sensory reactivity are significantly different between the two degrees of severity (medium versus high). Mann–Whitney tests showed that autistic children with higher severity of the autistic-like traits reported higher sensory reactivity compared to their counterparts in each dimension (TAS: U = 200.5; *p* < 0.001; TSS: U = 307.0; *p* = 0.001; MOV: U = 378.0 *p* = 0.016; USS: U = 292.0 *p* = 0.001; AF: U = 241.0; *p* < 0.001; LEW: U = 368.5; *p* = 0.012; VAS: U = 275.0; *p* < 0.001). Figure 1 shows the mean scores for each sensory profile dimension divided by the two degrees of severity of the autistic-like traits.

Examining the interaction of sensory reactivity dimensions x degree of severity of the autistic-like traits after controlling for the children’s ages and sex, the results confirmed the group comparison. The results showed that the degree of severity of the autistic-like traits significantly impacts the total score of sensory reactivity [F(1) = 27.842, *p* < 0.001, η^2^ = 0.535; η^2^p = 0.536]. This means that autistic children who reported a high degree of severity of autistic-like traits presented a higher sensory reactivity than their counterparts who reported a medium degree of severity.

No effect of the child’s age and sex was found.

**RQ2.**  *What dimensions of sensory reactivity are significantly different in the preschoolers and school-age autistic children? What is the interacting effect of the group (preschoolers versus schoolers) on the sensory reactivity after controlling for the child’s sex?*

**Figure 2:**
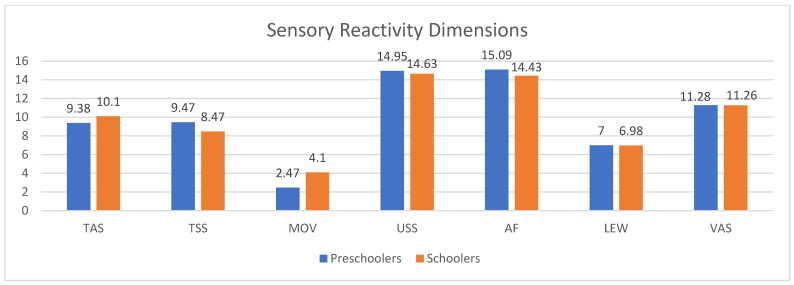
Sensory reactivity dimensions for preschoolers (blue) and schoolers (orange) groups. Note: TAS: Tactile Sensitivity; TSS: Taste/Smell Sensitivity; MOV: Movement; USS: Underresponsive/Seeks Sensation; AF: Auditory Filtering; LEW: Low Energy/Weak; VAS: Visual/Auditory Sensitivity.

Results of group comparisons showed that only the dimension of the sensory reactivity of Movement Sensitivity (MOV) is significantly different between preschoolers and schoolers; this means that the parents of the schoolers reported that their children are more anxious when their feet leave the ground, are scared of falling or heights, or dislike activities where the head is upside down compared to parents of preschoolers (MOV; U = 329.0; *p* = 0.027). Figure 2 shows the mean scores for each sensory profile dimension divided by the two groups of autistic children.

Examining the interaction of sensory reactivity dimensions x age groups (preschoolers versus schoolers) after controlling for the children’s sex, the results revealed no significant impact of the age group on the total score of the sensory reactivity [F(1) = 0.173, *p* = 0.678, η^2^ = 0.003; η^2^p = 0.000]. This means that autistic preschoolers and schoolers reported a similar degree of sensory reactivity.

No significant effect of the child’s sex was found.

## 4. Discussion

Although sensory reactivity has been introduced as a diagnostic criterion for autism (APA, 2013, 2022/2023), further investigation is needed to inform clinical protocols that address not only improvements in children’s developmental profiles but also their behavioural responses to sensory stimuli. The present study aimed to explore and describe sensory reactivity in a sample of preschool- and school-aged autistic children, to contribute to the existing literature and provide meaningful insights. To this end, the study was guided by two research questions. First, the study aimed to examine which dimensions of sensory reactivity differ significantly between autistic children reporting medium versus high levels of autistic-like trait severity, and to test the interaction effect between autistic-like trait severity (medium vs. high) and sensory reactivity total scores, while controlling for the child’s age and sex (RQ1). Second, the study aimed to investigate which dimensions of sensory reactivity differ between preschool-aged and school-aged autistic children, and to test the interaction effect of age group (preschoolers vs. school-aged children) on sensory reactivity total scores, controlling for the child’s sex (RQ2). As a preliminary step, associations between the children’s ages and the study’s variables were computed. Despite the heterogeneity of the sample, and in line with the previous literature (C. E. Robertson & Baron-Cohen, 2017), the results showed that autistic children reporting high levels of autistic-like trait severity displayed heterogeneous sensory reactivity profiles across multiple dimensions. In the current study, consistent with previous findings (Grzadzinski et al., 2020; Rossow et al., 2021), autistic-like trait severity was positively correlated with the severity of each sensory reactivity dimension. Thus, a greater frequency of autistic-like traits was associated with greater sensory reactivity. The lack of association between a child’s age and autistic-like traits may support the potential temporal stability of autistic-like traits (Barbaro & Dissanayake, 2017). Regarding the absence of an association between age and sensory reactivity severity, the results are not fully consistent with previous studies. For instance, Lane et al. (2022) found that autistic children aged 3–14.11 years reported a peak in sensory modulation symptoms during middle childhood, particularly in sensory sensitivity and avoidance. However, the small sample size and the wide age range in the present study may have influenced these findings, which therefore require further investigation. In this regard, future research could further examine sensory reactivity as a potential early behavioural predictor of autism, as suggested by longitudinal evidence (Grzadzinski et al., 2020).

With respect to the first research question (RQ1), examining the interaction between sensory reactivity and autistic-like trait severity (medium vs. high) while controlling for age and sex, the results indicated that autistic children with higher levels of autistic-like trait severity exhibited greater sensory reactivity than those with medium levels. This interaction effect was consistent with the preliminary group comparisons, showing higher sensory reactivity across all dimensions in children with more severe autistic-like traits. Although these findings diverge from previous studies (Lane et al., 2010, 2011), they invite reflection on how higher levels of autistic-like traits may influence children’s responses to environmental stimuli.

**Figure 3:**
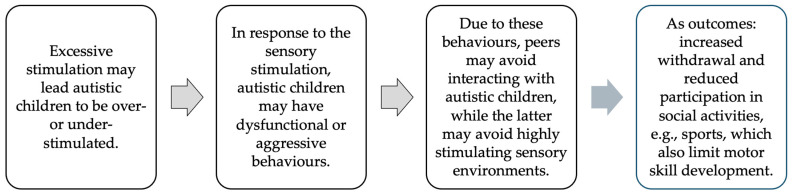
Cascade effect of sensory reactivity in autistic children on social and motor development.

Specifically, a cascade effect may emerge (see Figure 3). Autistic children’s challenges in social participation are primarily related to communicative and interactional challenges (Hirota & King, 2023). In addition, excessive sensory stimulation may lead children to either (1) respond in a dysfunctional or aggressive manner (Tomchek & Dunn, 2007), thereby increasing social alienation, or (2) avoid sensory exposure altogether, resulting in heightened social withdrawal (Little et al., 2015). In both cases, underestimating or neglecting sensory reactivity may have detrimental consequences for daily functioning, reducing participation and increasing difficulties in everyday activities (Little et al., 2015).

The second aim of the study (RQ2) examined the interaction between sensory reactivity and autistic-like traits across age groups (preschoolers vs. school-aged children), controlling for sex. The results revealed a non-significant interaction, suggesting that sensory reactivity and autistic-like trait severity do not differ substantially between preschool- and school-aged autistic children. Given the exploratory nature of the study, these findings may further support the potential longitudinal investigation of both autistic-like traits and sensory reactivity. Although the interaction between sensory reactivity and age group did not indicate a significant age effect, group comparisons revealed a significant difference in parental reports of movement sensitivity. Parents indicated that school-aged children (6–11 years) reacted more strongly than preschoolers (3–5 years) to rough play activities, such as those involving feet leaving the ground or inverted head positions. To our knowledge, similar findings have not been previously reported. A possible explanation may relate to emotional dysregulation during rough play, whereby older children may be more aware of the distressing effects of such activities, leading to heightened reactivity. It should be noted, however, that the descriptive nature of the study limits causal interpretations and generalizability, and future research using larger and more homogeneous samples is warranted. Particular attention should be paid to the role of age and sex as control variables. Regarding age, the present study found no significant effect on the interaction between sensory reactivity and autistic-like trait severity (RQ1). Evidence in the literature remains mixed, underscoring the need for further investigation. For example, meta-analyses by Ben-Sasson et al. (2009, 2019) reported age-related effects only for sensory-seeking behaviours, whereas other studies found no age-related differences in sensory processing (Leekam et al., 2007; Dwyer et al., 2020; Simpson et al., 2019; Tillmann et al., 2020). With respect to sex, no significant effects were found in either interaction [sensory reactivity × autistic-like trait severity (RQ1) and sensory reactivity × age group (RQ2)]. Although these findings may appear surprising given the well-documented sex differences in autism—with males often exhibiting more pronounced symptomatology than females (Hull et al., 2020; Levante et al., 2020a)—sex differences in sensory reactivity have been inconsistently reported. Some studies found no significant sex-related differences (Bitsika et al., 2018; Ben-Sasson et al., 2019; Tillmann et al., 2020), whereas others reported higher sensory scores in females across specific domains (Kumazaki et al., 2015; Lane et al., 2022). Given the sex imbalance in the present sample, these findings should be interpreted with caution.

In conclusion, the descriptive findings of the present study provide additional insights into the sensory experiences of autistic preschool- and school-aged children. Moreover, the knock-on effect associated with high levels of both autistic-like traits and sensory reactivity should be carefully considered in clinical practice. Assessing individual sensory reactivity profiles across dimensions may support professionals in developing targeted psychoeducational interventions and promoting sensory modulation. Similarly, helping parents identify and manage specific sensory triggers related to their children’s behavioural responses may be crucial in supporting daily functioning and coping with autistic features.

## 5. Strengths and Limitations

The strengths of the current study regard the investigation of the interaction between the severity of sensory reactivity, of autistic-like traits, and the children’s developmental stage (preschool- versus school-aged children). The insights provided by this study, although preliminary, are encouraging for clinical protocols. Given the early diagnosis of autism (Lecciso et al., 2019; Levante et al., 2020a; Levante et al., 2020b; Zwaigenbaum et al., 2015), being aware of the importance of taking into account a child’s sensory reactivity is pivotal, for example, to design an optimal environment for carrying out clinical protocols. In this vein, several authors (Camarata et al., 2020; Case-Smith et al., 2015; Harun et al., 2025) supported the debate on the best sensory-evidence intervention to improve children’s response to sensory stimuli. Nevertheless, a recent review (Harun et al., 2025) reported that while some studies showed improvements in sensory dimensions, the findings were inconsistent. Therefore, further rigorous research is needed to clarify the clinical value of clinical protocols in addressing sensory reactivity challenges.

A few limitations must be disclosed when examining our study in light of its results. A larger cohort of autistic children balanced according to age and sex would be needed to retest the interactions and the effects that emerged. Data regarding the diagnosis, the autistic-like traits, and sensory reactivity were parent-reported, and parents are a self-selected sample. Although the e-survey via social media allowed the scholars to collect data from a larger group of participants, it is a selection bias lacking control over the study’s variables, and a professional observation/assessment of the child is needed to test if the current results can be generalized. In addition, due to the small subsample size, the study did not explore the sensory profile in children reporting a low level of autistic traits. It could be useful to explore in future investigations. Although ANCOVA is robust to moderate assumption violations, nonparametric alternatives could provide a more conservative assessment in future studies. In addition, longitudinal studies are necessary to confirm whether the associations observed in the present paper remain consistent over time. Lastly, the study did not compare autistic children with other clinical conditions. Research in this vein is increasing (Caprì et al., 2020; Fabio et al., 2019, 2024; Mohammadhasani et al., 2020); however, future research is needed to investigate sensory reactivity in children with different neurodevelopmental conditions.

## 6. Research and Clinical Implications

Longitudinal studies exploring sensory reactivity in the autism context are scarce (Grzadzinski et al., 2020). Nevertheless, the promising insights provided by the current study support the design of further studies investigating whether sensory reactivity may vary by sex or age. Also, studies which deepen the variability of sensory reactivity according to autistic-like trait severity are needed; studies which test the effectiveness of clinical protocols mitigating sensory reactivity may also be useful.

For scholars, it may be interesting to explore more in-depth sensory reactivity in different autistic-like traits (low versus medium versus high) to detect more homogenous sensory profiles of autistic individuals based on specific sensory dimension difficulties. Subsequently, clinical protocols can be designed to address sets of sensory reactivity to each dimension.

Growing evidence on sensory reactivity in autistic children may support professionals in designing child-friendly environments that help mitigate difficulties related to sensory stimuli. For instance, sensory reactivity challenges may limit children’s participation in social and recreational activities (Parham et al., 2007), which in turn can hinder the development of adequate gross motor skills (Molloy et al., 2003). Awareness of autistic children’s sensory reactivity enables professionals to design targeted intervention protocols, such as those implemented in sport and physical activity contexts (Levante et al., 2023; Wen & Wu, 2025), aimed at promoting motor engagement while also fostering socio-emotional skills within environments that accommodate autism-related sensory needs.

In clinical settings, sensory reactivity-focused protocols may also provide meaningful support for families. When parents are trained to understand their children’s sensory reactivity profiles, they may be better equipped to manage behavioural responses to sensory stimuli, thereby reducing the likelihood of emotional dysregulation in the child and helping parents cope more effectively with parenting stress.

## Figures and Tables

**Table 1:** Frequencies of children in the severity classification.

	Low Level of Sensory Reactivity	Medium Level of Sensory Reactivity	High Level of Sensory Reactivity
Medium level of autistic-like trait	34	2	2
High level of autistic-like trait	13	7	10

**Table 2:** Correlations between the study’s variables.

	(1)	(2)	(3)	(4)	(5)	(6)	(7)	(8)
Autistic-like traits	0.534 ***	0.431 ***	0.315 **	0.407 ***	0.575 ***	0.344 **	0.514 ***	−0.013
TAS (1)		0.385 **	0.654 ***	0.710 ***	0.682 ***	0.543 ***	0.705 ***	0.019
TSS (2)			0.291 *	0.271	0.431 ***	0.234	0.359 **	−0.038
MOV (3)				0.389 **	0.434 ***	0.625	0.449 ***	0.186
USS (4)					0.801 ***	0.486 ***	0.626 ***	−0.133
AF (5)						0.431 ***	0.698 ***	−0.116
LEW (6)							0.633 ***	0.093
VAS (7)								0.072
Child’s age (8)								-

*** *p* < 0.001; ** *p* < 0.010; * *p* < 0.050. Note: TAS: Tactile Sensitivity; TSS: Taste/Smell Sensitivity; MOV: Movement; USS: Underresponsive/Seeks Sensation; AF: Auditory Filtering; LEW: Low Energy/Weak; VAS: Visual/Auditory Sensitivity.

## Data Availability

Data will be shared upon request.
